# Supplementary material for: Comparative efficacy of anti-vascular endothelial growth factor on diabetic macular edema diagnosed with different patterns of optical coherence tomography: A network meta-analysis
Source: PLoS One. 2024 Jun 7;19(6):e0304283. doi: 10.1371/journal.pone.0304283 (PMC11161126; doi:10.1371/journal.pone.0304283)
Supplement: S1 Fig — (DOCX) [file pone.0304283.s002.docx]

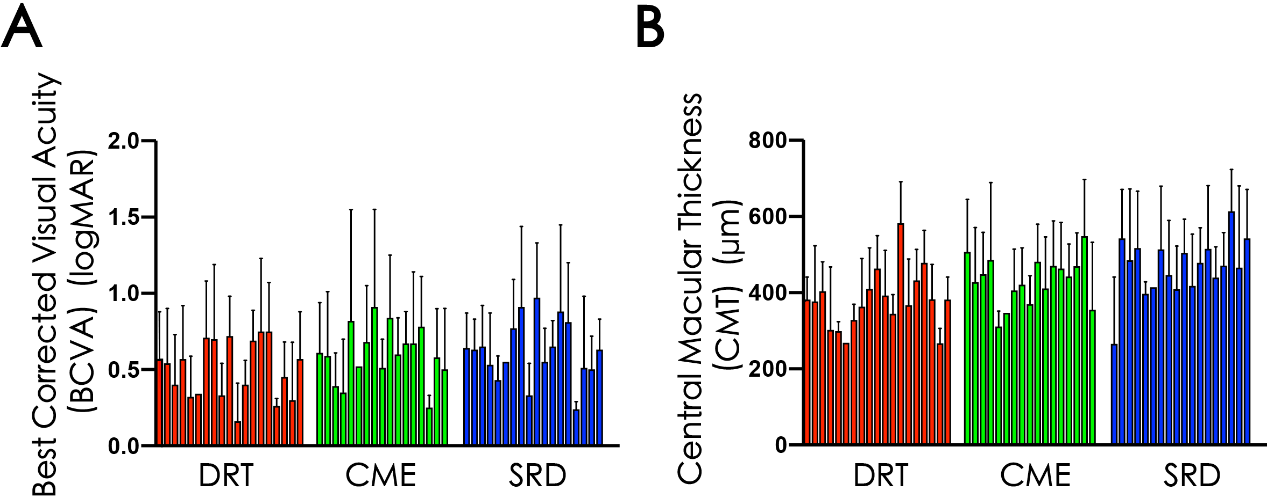
S1 Fig: Baseline values for BCVA (A) and CMT (B) in diabetes patients with different OCT patterns after anti-VEGF treatment.

Abbreviations: BCVA, best-corrected visual acuity; CME, cystoid macular edema; CMT, central macular thickness; DRT, diffuse retinal thickening; OCT, optical coherence tomography; SRD, serous retinal detachment; VEGF, vascular endothelial growth factor.
