# Supplementary material for: Comparative efficacy of anti-vascular endothelial growth factor on diabetic macular edema diagnosed with different patterns of optical coherence tomography: A network meta-analysis
Source: PLoS One. 2024 Jun 7;19(6):e0304283. doi: 10.1371/journal.pone.0304283 (PMC11161126; doi:10.1371/journal.pone.0304283)
Supplement: S5 Fig — (DOCX) [file pone.0304283.s006.docx]

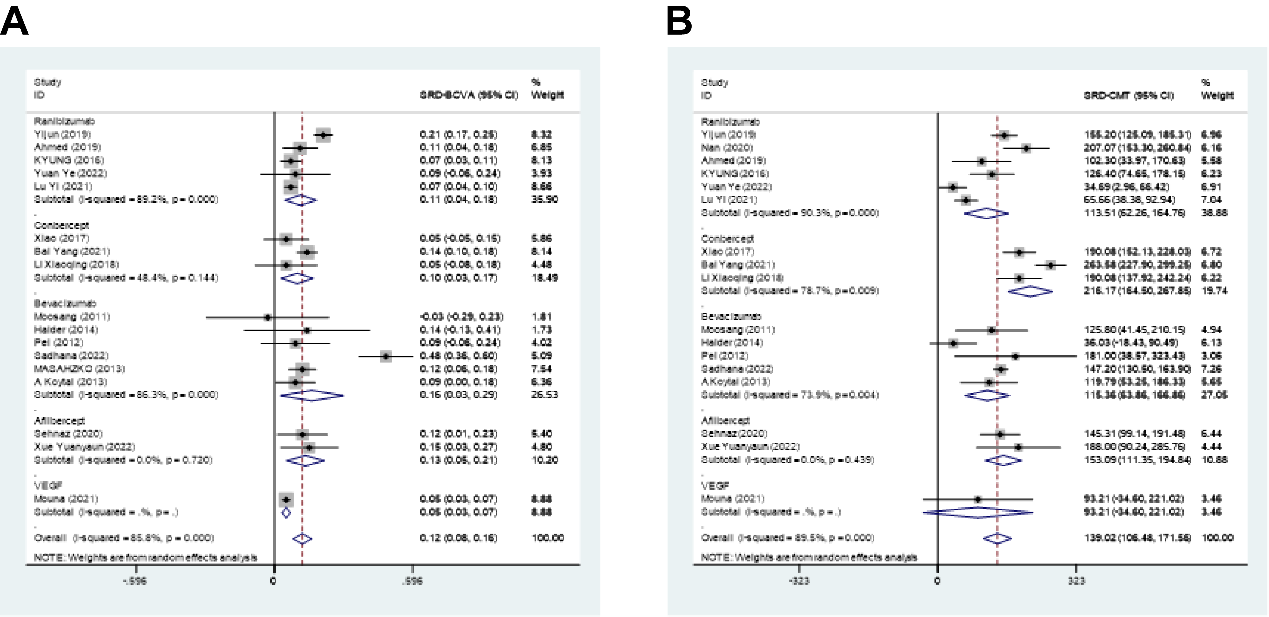


S5 Fig: Forest plot of BCVA (A) and CMT (B) outcomes in diabetes patients with SRD patterns among anti-VEGF drugs. Abbreviations: BCVA, best-corrected visual acuity; CMT, central macular thickness; CIs, confidence intervals; OCT, optical coherence tomography; SRD, serous retinal detachment; VEGF, vascular endothelial growth factor.
