# Supplementary material for: Comparative efficacy of anti-vascular endothelial growth factor on diabetic macular edema diagnosed with different patterns of optical coherence tomography: A network meta-analysis
Source: PLoS One. 2024 Jun 7;19(6):e0304283. doi: 10.1371/journal.pone.0304283 (PMC11161126; doi:10.1371/journal.pone.0304283)
Supplement: S1 Table — (DOCX) [file pone.0304283.s007.docx]

| Database | Search strategy | Hit Counts |
| --- | --- | --- |
| Pubmed (https://pubmed. ncbi.nlm.nih.gov/ advanced/) | #1 ALL fields=Macular edema cystoid or Macular Edema or Macular edema/CME or CMO/  #2 ALL fields=Diabets Mellitus or diabetes or Diabetic retinopathy/  #3 ALL fields= (vascular endothelial growth factor) or (VEGF) or bevacizumab or Mvasi or Bevacizumab-awwb or Avastin or ranibizumab or (RhuFab V2) or (V2, RhuFab) or Lucentis or conbercept or aflibercept or (VEGF Trap-regeneron) or (VEGF Trap-Eye) or (VEGF-Trap) or eylea or Zaltrap or (AVE 0005) or AVE0005 or (AVE-0005) or (AVE 005) or AVE005 or (AVE-005) or (ZIV-aflibercept)  #4 #1 AND #2 AND #3 | 2365 |
| Web of science (https://www.web ofscience.com/wo s/alldb/advanced search) | #1 TS= Macular edema cystoid or Macular Edema or Macular oedema/CME or CMO/  #2 TS=Diabets Mellitus or diabetes or Diabetic retinopathy/  #3 TS= (vascular endothelial growth factor) or (VEGF) or bevacizumab or Mvasi or Bevacizumab-awwb or Avastin or ranibizumab or (RhuFab V2) or (V2, RhuFab) or Lucentis or conbercept or aflibercept or (VEGF Trap-regeneron) or (VEGF Trap-Eye) or (VEGF-Trap) or eylea or Zaltrap or (AVE 0005) or AVE0005 or (AVE-0005) or (AVE 005) or AVE005 or (AVE-005) or (ZIV-aflibercept)  #4 #1 AND #2 AND #3 | 1937 |
| Embase (https://www.embase.com) | #1 Macular edema cystoid or Macular Edema or Macular oedema/CME or CMO/  #2 Diabet mellitus or diabetes or Diabetic retinopathy/  #3 (vascular endothelial growth factor) or (VEGF) or bevacizumab or Mvasi or Bevacizumab-awwb or Avastin or ranibizumab or (RhuFab V2) or (V2, RhuFab) or Lucentis or conbercept or aflibercept or (VEGF Trap-regeneron) or (VEGF Trap-Eye) or (VEGF-Trap) or eylea or Zaltrap or (AVE 0005) or AVE0005 or (AVE-0005) or (AVE 005) or AVE005 or (AVE-005) or (ZIV-aflibercept)  #4 #1 AND #2 AND #3 | 1029 |
| Medline(Ovid)  (https://ovidsp.dc2.ovid.com/) | #1 Macular edema cystoid or Macular Edema or Macular oedema/CME or CMO/  #2 Diabets Mellitus or diabetes or Diabetic retinopathy/  #3 (vascular endothelial growth factor) or (VEGF) or bevacizumab or Mvasi or Bevacizumab-awwb or Avastin or ranibizumab or (RhuFab V2) or (V2, RhuFab) or Lucentis or conbercept or aflibercept or (VEGF Trap-regeneron) or (VEGF Trap-Eye) or (VEGF-Trap) or eylea or Zaltrap or (AVE 0005) or AVE0005 or (AVE-0005) or (AVE 005) or AVE005 or (AVE-005) or (ZIV-aflibercept)  #4 #1 AND #2 AND #3 | 1524 |
| CNKI (https://www.cnk i.net/) | （FT=（糖尿病+黄斑水肿+糖尿病性黄斑水肿）AND FT=（抗VEGF+雷珠单抗+贝伐单抗+康博西普+阿柏西普））AND （SU=（糖尿病+黄斑水肿+糖尿病性黄斑水肿）OR TKA=（抗VEGF+雷珠单抗+贝伐单抗+康博西普+阿柏西普）） | 806 |
| WanFang (https://www.wan fangdata.com.cn/ ) | 全部：（糖尿病+黄斑水肿+糖尿病性黄斑水肿）and 全部（抗VEGF or 雷珠单抗 or 贝伐单抗 or 康博西普or 阿柏西普） | 742 |

S1 Table: The search strategy.
