# Supplementary material for: Comparative efficacy of anti-vascular endothelial growth factor on diabetic macular edema diagnosed with different patterns of optical coherence tomography: A network meta-analysis
Source: PLoS One. 2024 Jun 7;19(6):e0304283. doi: 10.1371/journal.pone.0304283 (PMC11161126; doi:10.1371/journal.pone.0304283)
Supplement: S2 Table — (DOCX) [file pone.0304283.s008.docx]

| Author | Year | Country | Intervention | Medication dosage | Treatment strategy | Average number of injections | Follow-up （month） | Baseline value of BCVA (LogMAR) | Baseline value of CMT (um) |
| --- | --- | --- | --- | --- | --- | --- | --- | --- | --- |
| **DRT group** | | | | | | | | | |
| Xiao-Qing Li | 2017 | China | conbercept | 0.5mg | 3+PRN | 3.69 | 12 | 0.57±0.31 | 381.64±59.60 |
| Moosang Kim | 2011 | Korea | bevacizumab | 1.25mg | 3+PRN | 3.67 | 12 | 0.54±0.36 | 377.1±145.9 |
| Sehnaz Ozcaliskan | 2020 | Turkey | aflibercept | 2.0mg | 3+PRN | 4.86 | 12 | 0.40±0.33 | 403.46±77.77 |
| Mi In Roh | 2010 | Korea | bevacizumab | 1.25mg | 1+PRN | 2.61 | 11.66 | 0.57±0.35 | 302.21±165.36 |
| Haider R.Cheema | 2014 | Ireland | bevacizumab | 1.25mg | 1+PRN | 2.05 | 6 | 0.32±0.268 | 298.8±25.03 |
| Muhammad Atif Mian | 2015 | Bahrain | bevacizumab | 1.25mg | 1+PRN | 1 | 1.5 | 0.34 | 268.2 |
| Nan-Ni Chen | 2020 | China | ranibizumab | 0.5mg | 3+PRN | 5.33 | 24 | 0.71±0.37 | 328.64±41.06 |
| Pei-Chen Wu | 2012 | China | bevacizumab | 1.25mg | 3+PRN | 3 | 3 | 0.70±0.49 | 362.9±126.63 |
| Ahmed T.AL Sayed | 2019 | Egypt | ranibizumab | 0.5mg | 3+PRN | 3 | 6 | 0.33±0.21 | 409.50±108.01 |
| Sadhana Sharma | 2022 | Nepal | bevacizumab | 1.25mg | 3+PRN | 3 | 3 | 0.72±0.26 | 463.05±86.31 |
| Mouna Al Saad | 2021 | Jordan | anti-VEGF | - | 3+PRN | 4.38 | 12 | 0.16±0.25 | 392.39±118.64 |
| KYUNG HOON SEO | 2016 | Korea | ranibizumab | 0.5mg | 3+PRN | 3.69 | 12 | 0.40±0.16 | 344.20±50.83 |
| MASAHIKO SHIMURA | 2013 | Japan | bevacizumab | 1.25mg | 1+PRN | 1 | 3 | 0.69±0.20 | 582.3±108.9 |
| A Koytak | 2013 | Tuekey | bevacizumab | 1.25mg | 3+PRN | 3 | 6 | 0.75±0.48 | 366.98±121.45 |
| Yuan Ye | 2022 | China | ranibizumab | 0.5mg | 5+PRN | 5 | 5 | 0.75±0.32 | 432.38±81.23 |
| Lu Yi | 2021 | China | ranibizumab | 0.5mg | 3+PRN | 3 | 3 | 0.26±0.05 | 478.32±85.21 |
| Bai Yang | 2021 | China | conbercept | 0.5mg | 3+PRN | 3.3 | 6 | 0.45±0.23 | 382.21±92.3 |
| Xue Yuanyuan | 2022 | China | aflibercept | 2mg | 3+PRN | 3 | 3 | 0.30±0.38 | 267.00±39.00 |
| Li Xiaoqing | 2018 | China | conbercept | 0.5mg | 2+PRN | 3.54 | 12 | 0.57±0.31 | 381.64±59.60 |
| **CME group** | | | | | | | | | |
| Xiao-Qing Li | 2017 | China | conbercept | 0.5mg | 3+PRN | 3.69 | 12 | 0.61±0.33 | 507.236±137.68 |
| Moosang Kim | 2011 | Korea | bevacizumab | 1.25mg | 3+PRN | 3.97 | 12 | 0.59±0.42 | 427.7±143.1 |
| Sehnaz Ozcaliskan | 2020 | Turkey | aflibercept | 2.0mg | 3+PRN | 5.17 | 12 | 0.39±0.22 | 448.50±110.05 |
| Mi In Roh | 2010 | Korea | bevacizumab | 1.25mg | 1+PRN | 2.61 | 11.66 | 0.35±0.35 | 485.46±203.41 |
| Haider R.Cheema | 2014 | Ireland | bevacizumab | 1.25mg | 1+PRN | 2.6 | 6 | 0.82±0.73 | 310.8±40.6 |
| Muhammad Atif Mian | 2015 | Bahrain | bevacizumab | 1.25mg | 1+PRN | 1 | 6 | 0.52 | 347.1 |
| Nan-Ni Chen | 2020 | China | ranibizumab | 0.5mg | 3+PRN | 5.33 | 24 | 0.68±0.37 | 405.66±108.53 |
| Pei-Chen Wu | 2012 | China | bevacizumab | 1.25mg | 3+PRN | 3 | 3 | 0.91±0.64 | 420.8±96.99 |
| Ahmed T.AL Sayed | 2019 | Egypt | ranibizumab | 0.5mg | 3+PRN | 3 | 6 | 0.51±0.19 | 369.70±74.337 |
| Sadhana Sharma | 2022 | Nepal | bevacizumab | 1.25mg | 3+PRN | 3 | 3 | 0.84±0.41 | 480.97±98.57 |
| KYUNG HOON SEO | 2016 | Korea | ranibizumab | 0.5mg | 3+PRN | 5.33 | 12 | 0.60±0.24 | 410.91±135.66 |
| MASAHIKO SHIMURA | 2013 | Japan | bevacizumab | 1.25mg | 1+PRN | 1 | 3 | 0.67±0.21 | 470.3±118.1 |
| A Koytak | 2013 | Tuekey | bevacizumab | 1.25mg | 3+PRN | 3 | 6 | 0.67±0.47 | 463.32±120.69 |
| Yuan Ye | 2022 | China | ranibizumab | 0.5mg | 5+PRN | 5 | 5 | 0.78±0.33 | 442.39±85.38 |
| Lu Yi | 2021 | China | ranibizumab | 0.5mg | 3+PRN | 3 | 3 | 0.25±0.08 | 469.74±86.98 |
| Bai Yang | 2021 | China | conbercept | 0.5mg | 3+PRN | 4.5 | 6 | 0.58±0.32 | 548.29±148.6 |
| Xue Yuanyuan | 2022 | China | aflibercept | 2mg | 3+PRN | 3 | 3 | 0.50±0.40 | 355.00±177.00 |
| Li Xiaoqing | 2018 | China | conbercept | 0.5mg | 2+PRN | 3.54 | 12 | 0.61 ±0.33 | 507.24 137.68 |
| **SRD group** | | | | | | | | | |
| Yijun Hu | 2019 | China | ranibizumab | 0.5mg | 3+PRN | 4.22 | 6 | 0.64±0.23 | 265.6±175.4 |
| Xiao-Qing Li | 2017 | China | conbercept | 0.5mg | 3+PRN | 3.69 | 12 | 0.63±0.20 | 542.04±128.86 |
| Moosang Kim | 2011 | Korea | bevacizumab | 1.25mg | 3+PRN | 3.79 | 12 | 0.65±0.27 | 485.1±187.1 |
| Sehnaz Ozcaliskan | 2020 | Turkey | aflibercept | 2.0mg | 3+PRN | 5.34 | 12 | 0.53±0.34 | 516.97±149.18 |
| Haider R.Cheema | 2014 | Ireland | bevacizumab | 1.25mg | 1+PRN | 2.6 | 6 | 0.43±0.16 | 397.15±31.05 |
| Muhammad Atif Mian | 2015 | Bahrain | bevacizumab | 1.25mg | 1+PRN | 1 | 6 | 0.55 | 414.2 |
| Nan-Ni Chen | 2020 | China | ranibizumab | 0.5mg | 3+PRN | 5.33 | 24 | 0.77±0.32 | 513.98±165.65 |
| Pei-Chen Wu | 2012 | China | bevacizumab | 1.25mg | 3+PRN | 3 | 3 | 0.91±0.53 | 446.3±143.58 |
| Ahmed T.AL Sayed | 2019 | Egypt | ranibizumab | 0.5mg | 3+PRN | 3 | 6 | 0.33±0.21 | 408.9±113.572 |
| Sadhana Sharma | 2022 | Nepal | bevacizumab | 1.25mg | 3+PRN | 3 | 3 | 0.97±0.36 | 504.54±88.72 |
| KYUNG HOON SEO | 2016 | Korea | ranibizumab | 0.5mg | 3+PRN | 5.09 | 12 | 0.55±0.22 | 417.42±136.19 |
| MASAHIKO SHIMURA | 2013 | Japan | bevacizumab | 1.25mg | 1+PRN | 1 | 3 | 0.65±0.17 | 478.0±92.2 |
| A Koytak | 2013 | Tuekey | bevacizumab | 1.25mg | 3+PRN | 3 | 6 | 0.88±0.57 | 515.05±165.83 |
| Yuan Ye | 2022 | China | ranibizumab | 0.5mg | 5+PRN | 5 | 5 | 0.81±0.39 | 439.72±80.29 |
| Lu Yi | 2021 | China | ranibizumab | 0.5mg | 3+PRN | 3 | 3 | 0.24±0.05 | 470.69±87.21 |
| Bai Yang | 2021 | China | conbercept | 0.5mg | 3+PRN | 4.9 | 6 | 0.51±0.47 | 613.59±110.2 |
| Xue Yuanyuan | 2022 | China | aflibercept | 2mg | 3+PRN | 3 | 3 | 0.50±0.22 | 465.50±214.50 |
| Li Xiaoqing | 2018 | China | conbercept | 0.5mg | 2+PRN | 3.54 | 12 | 0.63±0.20 | 542.04±128.86 |

S2 Table: Detailed treatment information and baseline values of BCVA and CMT in the DRT, CME, and SRD groups. Abbreviations: BCVA, best-corrected visual acuity; CME, cystoid macular edema; CMT, central macular thickness; DRT, diffuse retinal thickening; SRD, serous retinal detachment; VEGF, vascular endothelial growth factor.
