# Supplementary material for: Comparative efficacy of anti-vascular endothelial growth factor on diabetic macular edema diagnosed with different patterns of optical coherence tomography: A network meta-analysis
Source: PLoS One. 2024 Jun 7;19(6):e0304283. doi: 10.1371/journal.pone.0304283 (PMC11161126; doi:10.1371/journal.pone.0304283)
Supplement: S3 Table — (DOCX) [file pone.0304283.s009.docx]

| Variable Name | coef | Std.Err | Z | P | 95%CI |
| --- | --- | --- | --- | --- | --- |
| **DRT-BCVA group** | | | | | |
| Follow-up time | -0.010 | 0.007 | -1.553 | 0.144 | -0.026,0.004 |
| Average number of injections | 0.023 | 0.030 | 0.775 | 0.452 | -0.042,0.089 |
| Medication dosage | -0.044 | 0.048 | -0.926 | 0.371 | -0.148,0.059 |
| **DRT-CMT group** | | | | | |
| Follow-up time | -2.212 | 2.207 | -1.002 | 0.336 | -7.021,2.597 |
| Average number of injections | 11.115 | 11.482 | 0.968 | 0.352 | -13.901,36.131 |
| Medication dosage | -3.7959 | 18.23 | -1.745 | 0.107 | -71.500,7.908 |
| **CME-BCVA group** | | | | | |
| Follow-up time | -0.067 | 0.023 | 0 | 0.999 | -0.004,0.004 |
| Average number of injections | 0.025 | 0.014 | -1.342 | 0.180 | -0.003,0.053 |
| Medication dosage | -0.077 | 0.057 | -1.342 | 0.180 | -0.189,0.035 |
| **CME-CMT group** | | | | | |
| Follow-up time | -0.015 | 0.009 | -1.6460 | 0.124 | -0.035,0.005 |
| Average number of injections | -0.019 | 0.032 | 0.619 | 0.054 | -0.049,0.088 |
| Medication dosage | 0.041 | 0.050 | 0.821 | 0.427 | -0.067,0.149 |
| **SRD-BCVA group** | | | | | |
| Follow-up time | -0.015 | 0.009 | -1.646 | 0.124 | -0.035,0.005 |
| Average number of injections | 0.019 | 0.032 | 0.619 | 0.546 | -0.049,0.008 |
| Medication dosage | 0.041 | 0.50 | 0.821 | 0.427 | -0.067,0.149 |
| **SRD-CMT group** | | | | | |
| Follow-up time | 2.96 | 4.020 | 0.737 | 0.474 | -5.721,11.650 |
| Average number of injections | 8.45 | 22.730 | 0.372 | 0.716 | -40.646,57.565 |
| Medication dosage | 11.742 | 30.230 | 0.388 | 0.704 | -53.566,77.050 |

S3 Table: Meta-regression analysis on BCVA and CMT outcomes across DRT, CMT, and SRD patient groups. Abbreviations: BCVA, best-corrected visual acuity; CME, cystoid macular edema; CMT, central macular thickness; DRT, diffuse retinal thickening; SRD, serous retinal detachment; coef, coefficient; Std.Err, standard error.
